# Risk Model–Guided Clinical Decision Support for Suicide Screening: A Randomized Clinical Trial

**DOI:** 10.1001/jamanetworkopen.2024.52371

**Published:** 2025-01-03

**Authors:** Colin G. Walsh, Michael A. Ripperger, Laurie Novak, Carrie Reale, Shilo Anders, Ashley Spann, Jhansi Kolli, Katelyn Robinson, Qingxia Chen, David Isaacs, Lealani Mae Y. Acosta, Fenna Phibbs, Elliot Fielstein, Drew Wilimitis, Katherine Musacchio Schafer, Rachel Hilton, Dan Albert, Jill Shelton, Jessica Stroh, William W. Stead, Kevin B. Johnson

**Affiliations:** 1 Department of Biomedical Informatics, Vanderbilt University Medical Center, Nashville, Tennessee; 2 Department of Medicine, Vanderbilt University Medical Center, Nashville, Tennessee; 3 Department of Psychiatry and Behavioral Sciences, Vanderbilt University Medical Center, Nashville, Tennessee; 4 Department of Biostatistics, Vanderbilt University Medical Center, Nashville, Tennessee; 5 Department of Neurology, Vanderbilt University Medical Center, Nashville, Tennessee; 6 Department of Psychology, Vanderbilt University Medical Center, Nashville, Tennessee; 7 Department of Psychiatry and Sleep Medicine, Stanford University, Palo Alto, California; 8 Health Information Technology, Vanderbilt University Medical Center, Nashville, Tennessee; 9 Department of Biostatistics, Epidemiology and Informatics, University of Pennsylvania, Philadelphia; 10 Department of Computer and Information Sciences, University of Pennsylvania, Philadelphia

## Abstract

**Importance:**

Suicide prevention requires risk identification, intervention, and follow-up. Traditional risk identification relies on patient self-reporting, support network reporting, or face-to-face screening. Statistical risk models have been studied and some have been deployed to augment clinical judgment. Few have been tested in clinical practice via clinical decision support (CDS). Barriers to effective CDS include potential alert burden for a stigmatized clinical problem and lack of data on how best to integrate scalable risk models into clinical workflows.

**Objective:**

To evaluate the effectiveness of risk model–driven CDS on suicide risk assessment.

**Design, Setting, and Participants:**

This comparative effectiveness randomized clinical trial was performed from August 17, 2022, to February 16, 2023, in the Department of Neurology across the divisions of Neuro-Movement Disorders, Neuromuscular Disorders, and Behavioral and Cognitive Neurology at Vanderbilt University Medical Center, an academic medical center in the US Mid-South. Patients scheduled for routine care in those settings were randomized at visit check-in. Follow-up was completed March 16, 2023, and data were analyzed from April 11 to July 24, 2023. Analyses were based on intention to treat.

**Interventions:**

Interruptive vs noninterruptive CDS to prompt further suicide risk assessment using a real-time, validated statistical suicide attempt risk model. In the interruptive CDS, an alert window via on-screen pop-up and a patient panel icon were visible simultaneously. Dismissing the alert hid it with no effect on the patient panel icon. The noninterruptive CDS showed the patient panel icon without the pop-up alert. When present, the noninterruptive CDS displayed “elevated suicide risk score” in the patient summarization panel. Hovering over this icon resulted in a pop-up identical to the interruptive CDS.

**Main Outcomes and Measures:**

The main outcome was the decision to assess risk in person. Secondary outcomes included rates of suicidal ideation and attempts in both treatment arms and baseline rates of documented screening during the prior year. Manual medical record review of every trial encounter was used to determine whether suicide risk assessment was subsequently documented.

**Results:**

A total of 561 patients with 596 encounters were randomized to interruptive or noninterruptive CDS in a 1:1 ratio (mean [SD] age, 59.3 [16.5] years; 292 [52%] women). Adjusting for clinician cluster effects, interruptive CDS led to significantly higher numbers of decisions to screen (121 of 289 encounters [42%]) compared with noninterruptive CDS (12 of 307 encounters [4%]) (odds ratio, 17.70; 95% CI, 6.42-48.79; *P* < .001) and compared with the baseline rate the prior year (64 of 832 encounters [8%]). No documented episodes of suicidal ideation or attempts occurred in either arm.

**Conclusions and Relevance:**

In this randomized clinical trial of interruptive and noninterruptive CDS to prompt face-to-face suicide risk assessment, interruptive CDS led to higher numbers of decisions to screen with documented suicide risk assessments. Well-powered large-scale trials randomizing this type of CDS compared with standard of care are indicated to measure effectiveness in reducing suicidal self-harm.

**Trial Registration:**

ClinicalTrials.gov Identifier: NCT05312437

## Key Points

**Question:**

In settings without universal suicide risk screening, is interruptive clinical decision support (CDS) with an on-screen pop-up more effective than noninterruptive CDS in prompting in-person risk assessment at the point of care for patients predicted by a statistical model to be at high risk of a suicide attempt?

**Findings:**

In this randomized clinical trial of 561 participants with 596 clinician encounters, interruptive CDS was significantly more effective at prompting in-person assessment than noninterruptive CDS and more effective compared with baseline documented screening rates the prior year.

**Meaning:**

These results suggest that well-powered large-scale trials randomizing interruptive CDS compared with standard of care are indicated to measure effectiveness in reducing suicidal thoughts and behaviors in the context of alert burden and capacity constraints.

## Teaser

This randomized clinical trial tests whether interruptive clinical decision support (CDS) prompted more frequent in-person suicide risk assessments than noninterruptive CDS and increased in-person screening rates compared with the prior year.

## Introduction

Improving suicide prevention requires appropriate risk identification, prognostication, and effective intervention. Risk identification combines clinical judgment, validated screening instruments, and a growing cadre of validated statistical models.^1,2^ Computational risk estimation might be suited to prompt further suicide risk assessment and/or intervention, but effectiveness of model-driven clinical decision support (CDS) systems in suicide prevention is understudied.^2,3,4^ The most prominent evaluation before and after a preventive outreach program, REACH VET (Recovery Engagement and Coordination for Health–Veterans Enhanced Treatment), remains an exemplar to date, though no such system has been studied via a randomized clinical trial (RCT) to our knowledge.^5^

Screening within health care encounters remains a priority for the field, given the incidence of suicide shortly after such encounters; in some cases, suicidal self-harm occurred the same day in which at-risk patients were seen.^6^ Screening remains particularly relevant in primary and non–mental health specialty care, the most common point of contact in the year prior to death due to suicide, with 77% of those who die by suicide seen in primary care in the preceding year.^7^ The US Preventive Services Task Force recommends screening for suicide in adult primary care settings,^8^ but universal standards on practice-wide screening are lacking.^7^

Because of gaps in reliable screening, much attention has been given to developing instruments and to automating or semiautomating risk prognostication to bolster risk identification. Traditional suicide risk prognostication relies on clinical judgment guided by validated instruments like the Patient Health Questionnaire,^9^ the Columbia Suicide Severity Rating Scale (CSSRS),^10^ the Ask Suicide-Screening Questions toolkit,^11^ and others.^12,13^ In the last decade, a myriad of validated statistical models have been published to improve suicide estimation.^1^ These include Army STARRS (Study to Assess Risk and Resilience in Servicemembers),^14,15,16^ REACH VET,^5,17,18^ the Mental Health Research Network,^19,20^ and many more.^21,22,23,24,25,26^ Recent research suggests that statistical modeling combined with face-to-face screening outperform either alone.^27^

To enable prevention, predictive models must be actualized through tools like CDS. Prior literature outside of suicide research has examined forms of CDS such as interruptive (eg, alerts) and noninterruptive (eg, static icons or visual cues) to inform contact isolation decisions (physician decisions to order transmission-based precautions to prevent contact transmission of infectious diseases during hospitalization),^28^ laboratory alerts,^29^ and blood transfusion.^30^ While interruptive CDS tends to be more effective in driving behavior, this question has not been studied via RCT in suicide-preventive workflows, to our knowledge. Also, given significant concerns around false-positive findings in suicide screening,^31,32^ demonstrating adequate performance of a noninterruptive CDS would support implementing a less burdensome and stigmatizing alert.^33,34^

Our team has previously validated, replicated, and prospectively “silently” tested (ie, running the model in real-time in production systems without alerting of any kind to ensure ongoing accuracy and validity) an electronic health record (EHR)–based suicide risk model.^27,35,36^ Herein, we report design and evaluation of CDS driven by that model to prompt suicide risk assessment within health care encounters in settings that do not conduct universal screening. A non–behavioral health setting with increased suicide risk^37^ and variable suicide prevention workflows, ambulatory neurology clinics, serve as the trial setting. Unlike high-risk settings such as the emergency department, ambulatory neurology clinics do not have universal screening protocols in all sites. Despite the absence of these protocols, some patients in these settings have increased suicide risk, such as those with movement disorders and inherited disorders like Huntington disease.^38,39^

The designs of the CDS and the research protocol are informed by human-centered design (HCD),^40^ a framework to evaluate appropriate CDS alerts and responses,^41^ and a deployment framework for clinical artificial intelligence.^42^ We conduct a comparative effectiveness RCT of our risk model–prompted CDS, assessing the interruptive and noninterruptive designs^28,43,44^ to prompt suicide risk assessment within clinical encounters. We hypothesize that the interruptive CDS arm would lead to higher rates of in-person suicide risk assessment primarily compared with the noninterruptive CDS arm and secondarily compared with the prior year.

The primary aim of this study was to test whether interruptive CDS prompted more frequent in-person suicide risk assessment than noninterruptive CDS, as has been shown in other clinical domains.^28,29,30,43^ The secondary aim tested whether CDS increased in-person screening rates compared with the prior year.

## Methods

This 2-arm RCT uses a validated risk model to prompt suicide-preventive CDS at the start of routine health care encounters.^27,35,36,45^ Waiver of consent was requested and approved by the Vanderbilt University Medical Center institutional review board. This request was based on concern for introducing bias into clinical encounters in which clinicians might credibly disagree with the CDS and decide not to assess suicide risk, which otherwise might be prompted by the consent process itself. This RCT protocol (found in Supplement 1) adhered to the Standard Protocol Items: Recommendations for Interventional Trials (SPIRIT) guidelines; the report followed the Consolidated Standards of Reporting Trials (CONSORT) for reporting clinical trials.

### Study Setting

The study settings in this RCT include ambulatory neurology clinics across the divisions of Neuro-Movement Disorders, Neuromuscular Disorders, and Behavioral and Cognitive Neurology at Vanderbilt University Medical Center, an academic medical center in the US Mid-South. The patients typical of this setting include those with a range of general neurological concerns ranging from headache to neurocognitive, memory, and movement disorders. The trial was conducted from August 17, 2022, through February 16, 2023, with a 30-day follow-up period ending March 16, 2023, to assess for secondary outcomes (ie, documented episodes of suicidal ideation or suicide attempt following study encounters). Race and ethnicity were determined by demographic tables in the electronic health record. We recorded these data because, despite race being a social construct and a push not to collect these data in current analyses, historical rates of suicide by recorded race have been fraught with disparities. For example, Bray et al found that rates of suicide among Black residents of Maryland increased during the COVID-19 pandemic despite the perception that suicide rates did not change (they decreased for White residents).^46^ Thus, we deemed it appropriate to at least evaluate metrics by race and ethinicity with the intent to develop algorithmovigilant systems, until better definitions exist that overcome the failings of race.

### Intervention (CDS) Design

We codesigned the CDS with neurologists through multiple meetings with volunteer clinicians in the study clinics. Our HCD experts (L.N., C.R., and S.A.) with our EHR physician builder (A.S.) designed interruptive and noninterruptive versions of the CDS. In the interruptive CDS, an alert window (best practice alert) and a patient panel icon were visible simultaneously. Dismissing the alert hid it with no effect on the patient panel icon. eFigure 1 in Supplement 2 includes all CDS visualizations.

The noninterruptive CDS used a summarization panel for patient-level data foundational to the EHR interface (Epic Systems Corporation storyboard). When relevant, the noninterruptive CDS displayed “elevated suicide risk score” in the patient summarization panel (shown in eFigure 1 in Supplement 2). Hovering over this icon resulted in a pop-up identical to the interruptive CDS. Clicking that pop-up permitted clinicians to act on the alert identically to interaction in the interruptive CDS arm. Both versions of the CDS included a feedback tool for unprompted, free-text comments from users built directly into the user interface (shown in eFigure 1 in Supplement 2).

**Figure 1.**
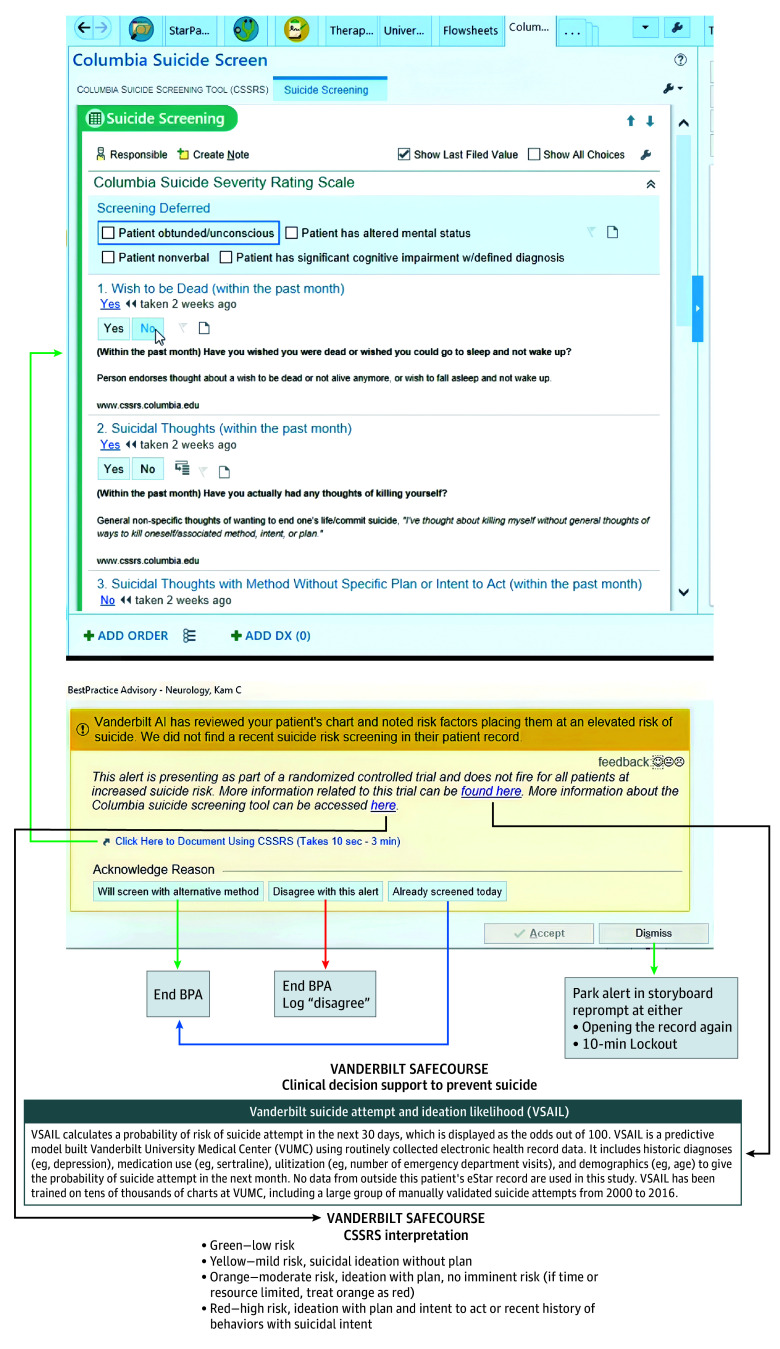
Clinical Decision Support Logic Detailed views of each interface are given in eFigure 1 in Supplement 2.

During HCD focus groups,clinicians requested a way to better document suicide screening assessments within encounters. In response, we developed a customized form (shown in eFigure 2 in Supplement 2) using the CSSRS, our medical center’s chosen instrument for universal screening mandated by the Joint Commission.^47^ Overall CDS logic links interactions directly to trial outcomes (Figure 1).

### Randomization and Masking

During patient check-in or registration for an encounter in the clinic or over telehealth, our validated risk model calculates 30-day suicide attempt risk (probability) using operational data across diagnoses, medications, visit utilization, and demographics.^35,36^ These probabilities are transmitted to flowsheets in the EHR to prompt CDS. One-to-one randomization occurred for all patients with predicted risk above or equal to 2%, a threshold chosen from prior validation.^36^ In that prior work, this optimal threshold was determined as the risk probability above which all predictions were in the highest-risk tier with concomitantly the highest concentration of suicide risk. Of note, this threshold applied in settings without universal screening, whereas the same threshold in universal screening settings was 3%.^36^ Randomization was conducted directly within the EHR with half of encounters randomized to interruptive CDS and the remainder to noninterruptive CDS. The intervention itself reflects randomization status, that is, interruptive or not, making masking or blinding the intervention infeasible.

### Trial Eligibility and Inclusion

As this trial was pragmatic in design, all patients appearing for routine care in study settings were eligible. Patients already scheduled for neurological care were enrolled in this trial.

### Ethics Approval

Ethicists were represented on our study team throughout the study design period. The study team met with the Vanderbilt University Medical Center Office of Legal Affairs prior to trial start, given the sensitive nature of suicide prevention to avoid unintended liability risks to clinicians.

### Primary Outcome and Sample Size

The decision to assess suicide risk in person through CDS interaction served as the primary trial outcome. The primary outcome was recorded via direct interaction with study CDS (see Figure 1).

With approximately 15 patients per week per arm estimated from silent validation,^36^ we hypothesized that interruptive CDS would be more effective at prompting in-person suicide risk assessment than noninterruptive CDS (20% compared with 5%). Thus, we needed at least 75 patients in each arm to achieve 90% power with 5% probability of type I error.

### Secondary Outcomes

Secondary trial outcomes included rates of 30-day episodes of suicidal ideation, suicide attempt, rates of documented suicide risk assessment in clinical notes, psychiatric hospitalization, or emergency department utilization related to mental illness and/or suicide risk. We ascertained suicidal ideation and attempts with any documented diagnostic codes or through medical record review. Diagnostic codes in the *International Statistical Classification of Diseases, 10th Revision*, have been shown to have high positive predictive value in prior research, including positive predictive value of 0.85 for suicide attempt and 0.96 for suicidal ideation.^48^ We ascertained hospitalization or emergency department utilization with EHR health care encounter data.

We assessed documentation via medical record review of every trial encounter by 2 members of the study team (J.K. and K.R.), with adjudication when needed by the principal investigator (C.G.W.). During the medical record review, annotators recorded the presence or absence of documented suicide risk assessments and justified clinical text (seed terms, eg, “denied SI [suicidal ideation]”).

### Comparator Analysis Before and After Program Intervention

Because this RCT compares CDS effectiveness, a standard of care comparator for patients with similar predicted risk was assessed from August 17, 2021, to February 16, 2022, 1 year earlier than the RCT. We conducted an identical analysis of notes during this earlier period using the same seed terms extracted during RCT medical record review above (see eMethods in Supplement 2 for seed terms).

### Thematic Analysis

Free text comments entered by clinicians into our CDS were reviewed manually and separately by 3 members of the study team (C.G.W., L.N., and S.A.) for emergent themes and patterns. Comments are labeled by theme independently and then compared across reviewers for consensus. In instances of disagreement between 2 reviewers, the third reviewer adjudicated the final label for that comment.

### Training, Outreach, and Education

To increase engagement, acculturate physicians to the RCT, and begin CDS education, our study team engaged clinical teams first through division seminars for the Department of Neurology. Department-wide and targeted email supplemented this effort to increase awareness of the upcoming trial. Educational materials were prepared and disseminated, including an instructional video with demonstrations of both interruptive and noninterruptive CDS. Materials were distributed via email to physicians in each trial site. Additionally, materials were stored in a secured document repository and accessible directly from links built into the CDS itself (eFigure 1 in Supplement 2).

### Protocol Deviations and Safety and Adverse Events

We monitored the trial protocol throughout the study, including monthly check-ins with clinical sites and feedback tools built into the CDS. The study team (C.G.W., M.A.R., L.N., C.R., J.K., and K.R.) reviewed trial progress monthly and shared study personnel contact information broadly with participating sites.

### Statistical Analysis

We used χ^2^ test statistics to support primary hypothesis testing. We analyzed baseline study characteristics with descriptive statistics and pooled *P* values. The Cohen κ statistic measured interrater agreement in medical record review. We summarized baseline variables in counts and frequencies or mean and SD for categorical or continuous variables, respectively. We used χ^2^ and 2-sample *t* tests to compare variables between the 2 arms. The level of significance was *P* < .05, and the tests were 2 sided. The primary study outcome, decision to screen, and one of the secondary outcomes, documentation of suicide risk assessment, were assessed with logistic regression models using the Huber-White method to adjust for potential cluster effects by clinician.^49^ The secondary study outcome, screening rates before and after the intervention, are assessed with descriptive statistics. Statistical analyses were conducted April 11 to July 24, 2023, using R, version 4.2.1 (R Project for Statistical Computing).

## Results

### Study Sample

**Figure 2.**
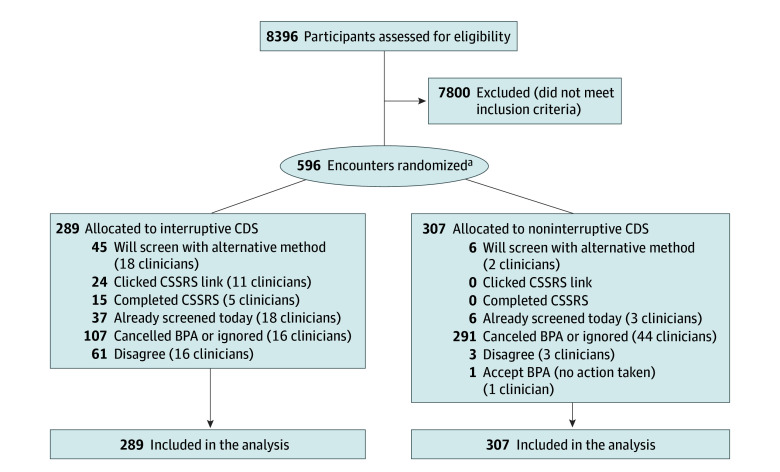
Participant Flow Diagram BPA indicates best practice alert; CDS, clinical decision support; and CSSRS, Columbia Suicide Severity Rating Scale. ^a^Includes 561 patients with first encounters and 72 clinicians.

From August 16, 2022, through February 16, 2023, our study randomized 596 of 7732 total encounters (8%) in RCT settings (Figure 2). The trial ended as scheduled with sufficient sample sizes to assess primary outcome differences per arm. The randomized encounters involved 561 of 6062 total patients (9%). Seventy-one clinicians participated in the trial receiving either interruptive or noninterruptive CDS, including 24 attendings, 26 resident physicians, 6 fellows, 6 nurse practitioners, 5 psychologists, 3 genetic counselors, and 1 physician assistant. The baseline study characteristics at the first study encounter for patients seen in this period are shown (Table). Of the 561 patients included, mean (SD) age was 59.3 (16.5) years; 269 (48%) were men and 292 (52%) were women. Ten patients (2%) were American Indian or Alaska Native, Asian, Hispanic, multiracial, declined to answer, or other; 17 (3%), Black; 306 (55%), White; and 228 (41%), unknown race or ethnicity. 

**Table.** Baseline Study Characteristics

Characteristic	Study arm, No. (%)	
	Interruptive CDS with >2% predicted risk	Noninterruptive CDS with >2% predicted risk
**Encounters**		
All (n = 596)	289 (100)	307 (100)
Division within Department of Ambulatory Neurology		
Behavioral and Cognitive Neurology	76 (26)	90 (29)
Neuro-Movement Disorders	112 (39)	121 (39)
Neuromuscular Disorders	101 (35)	96 (31)
**Patients at first trial encounter**		
All (n = 561)	267 (100)	294 (100)
Age, mean (SD), y	58.8 (16.5)	59.8 (16.4)
Coded sex		
Men	137 (51)	132 (45)
Women	130 (49)	162 (55)
Coded race and ethnicity		
American Indian or Alaska Native, Asian, Hispanic, other, multiracial, or declined	6 (2)	4 (1)
Black	8 (3)	9 (3)
White	137 (51)	169 (57)
Unknown	116 (43)	112 (38)
Suicide risk probability, mean (SD)^a^	0.034 (0.012)	0.033 (0.013)

^a^ Calculated using operational data across diagnoses, medications, visit utilization, and demographics.

### Primary Trial Outcome

Of 289 encounters in the interruptive CDS arm, 121 (42%) resulted in clinicians electing to screen either through the CSSRS or another assessment of their choosing. Of 307 encounters in the noninterruptive CDS arm, 12 (4%) led to screening with the CSSRS or other assessment. Accounting for cluster effects by individual clinicians, the interruptive CDS prompted higher rates of in-person screening compared with noninterruptive CDS (odds ratio, 17.70; 95% CI, 6.42-48.79; *P* < .001), consistent with the study alternative hypothesis. Analyses were based on intention to treat.

### Secondary Trial Outcomes

**Figure 3.**
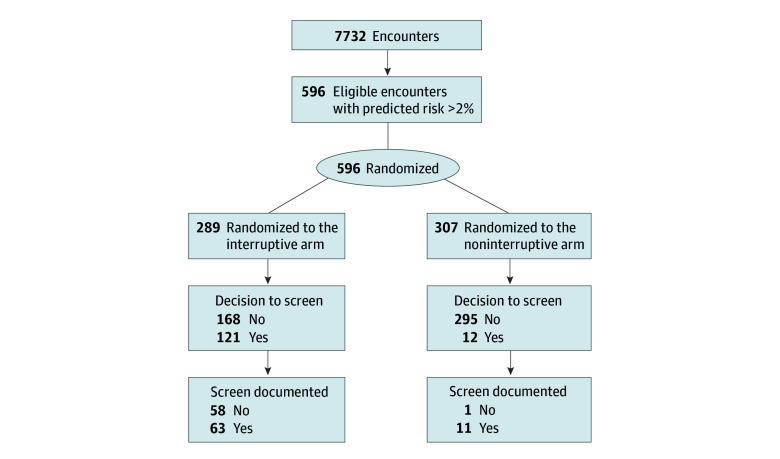
Flowchart of Trial Outcomes by Arm

While the proportion of documented risk assessments among those noting the decision to screen was higher for clinicians in the noninterruptive arm (11 of 12 [92%]) than in the interruptive arm (63 of 121 [52%]), the interruptive CDS was associated with more frequent documentation of suicide risk assessment (63 of 289 encounters [22%] compared with 11 of 307 [4%]; *P* < .001). Figure 3 indicates decisions to screen (primary outcome) and documentation rates (secondary) by trial arm.

No clinical secondary events occurred following study encounters, including 30-day episodes of suicidal ideation, 30-day episodes of suicide attempt, psychiatric hospitalizations, or emergency department utilization related to mental illness and/or suicide risk. The 2 reviewers had excellent agreement in independent, manual medical record review to confirm documentation of suicide risk assessment (Cohen κ = 0.9; goal, >0.8). No adverse safety events or protocol deviations were reported throughout the trial. N documented episodes of 30-day suicidal ideation, suicide attempt, emergency department utilization for mental illness, or psychiatric hospitalizations occurred in either arm.

### Analysis Before Intervention

We assessed a standard of care comparator from August 2021 through February 2022, 1 year before the intervention. Compared with a study-period rate of 22% (63 of 289) encounters, the same clinical settings had a baseline suicide risk assessment rate of 8% (64 of 832 encounters) in the prior year.

### Thematic Analysis of Clinician Comments

Through the design of the CDS, clinicians were able to share comments on use of the alerts in practice. Forty comments were entered of all 596 study encounters (7%; 13 in the noninterruptive and 27 in the interruptive arm). Comments are shared in the eTable in Supplement 2). The major themes included whether or not a patient was screened and whether the result of that screen was negative (32 of 40 comments); whether the alert was inappropriate for the patient (4 of 40 comments); and whether the screening was deferred for the patient (2 of 40 comments).

## Discussion

This pragmatic RCT compared the effectiveness of 2 versions of CDS prompted by real-time statistical risk modeling in the clinic. In line with the primary trial aim, interruptive CDS prompted 18-fold higher rates of decision to screen compared with noninterruptive CDS. Interruptive CDS prompted nearly 3-fold higher rates of documented screening compared with the prior year (22% compared with 8%, a secondary study aim). For the secondary trial aim, noninterruptive CDS showed a higher proportion of documented assessment following a decision to screen. No 30-day suicide events followed study encounters throughout the trial.

This RCT builds on understanding of effectiveness of forms of CDS to drive clinical decisions. Interruptive CDS tends to be more effective in prompting behavior. This finding has been shown in diverse settings including medication management for heart failure,^43^ contact isolation practices in the emergency department,^28^ and Patient Health Questionnaire–9 administration in primary care clinics.^50^ Disadvantages of interruptive CDS, especially alert fatigue, counterbalance its effectiveness.^51,52^ Clinicians prefer passive, noninterruptive CDS even while acknowledging they might not be seen nor used as often as interruptive prompts.^53^ We note that regardless of the form of CDS, using a validated statistical model to prompt CDS reduced the potential burden of screening to only 8% of all 7732 encounters in trial settings.

As a single-center RCT, this study has implications for trial design using artificial intelligence–driven tools, preventive workflows in ambulatory settings, and CDS. Suicide remains a stigmatized area with risk management concerns, but this study provides evidence how trial designs might rigorously test novel approaches without sacrificing equipoise. CDS designs in ambulatory settings, high throughput clinical environments where encounters might be brief, might leverage various approaches based on priorities of those clinical areas. Where recall matters most, noninterruptive CDS permits visualization of risk information in context of where clinicians are delivering care without interrupting their workflow. Where rates of screening are deemed too low, interruptive CDS might be more effective at prompting behavioral change. Finally, an HCD in CDS facilitated tool uptake and candid feedback from clinicians that should inform iterative improvement of CDS delivered to the point of care.

### Limitations

Limitations of this RCT include potential leakage of suicide risk assessment that might have occurred regardless of the presence of CDS. The RCT was not powered to detect changes in rates of suicide attempts or deaths (neither of which occurred during the RCT). Clinicians participating in the trial might have used the tools more frequently due to performance bias, though the noninterruptive CDS resulting in a lower decision-to-screen rate than baseline in the prior year counterbalances this point. Our team was unable to determine whether participating clinicians used training materials via email; future research of the impact of design of training materials is indicated. CDS iterative design including integration of treatment recommendations as well as larger-scale RCTs of this type of CDS should be foci of future work.

## Conclusions

In this RCT, a validated predictive model prompted randomization to 2 forms of CDS prevention. Interruptive CDS outperformed noninterruptive CDS in prompting in-person assessments. The predictive modeling trigger reflects the need to precisely prompt conversations about suicide risk, a clinical priority that remains rare at the scale of a health system. A larger-scale trial focused on the effectiveness of the overall system powered sufficiently against standard of care to reduce suicide events is indicated. Future research might also further iterate CDS design, test these systems in more diverse settings, and integrate preventive recommendations beyond risk assessment.

## Notes

Supplement 1.
Trial Protocol

Supplement 2. **eFigure 1.** Interruptive and Noninterruptive CDS User Interfaces (Fake Patient Data Shown) **eFigure 2.** The CSSRS Custom Form (Excerpt) Developed for This RCT **eMethods.** Seed Terms Derived From RCT Medical Record Review **eTable.** Comments Input to CDS by Theme

Supplement 3.
Data Sharing Statement
