# Supplementary material for: Risk Model–Guided Clinical Decision Support for Suicide Screening: A Randomized Clinical Trial
Source: JAMA Netw Open. 2025 Jan 3;8(1):e2452371. doi: 10.1001/jamanetworkopen.2024.52371 (PMC11699529; doi:10.1001/jamanetworkopen.2024.52371)
Supplement: Supplement 2. — eFigure 1. Interruptive and Noninterruptive CDS User Interfaces (Fake Patient Data Shown) eFigure 2. The CSSRS Custom Form (Excerpt) Developed for This RCT eMethods. Seed Terms Derived From RCT Medical Record Review eTable. Comments Input to CDS by Theme [file jamanetwopen-e2452371-s002.pdf]

Supplementary Online Content

Walsh CG, Ripperger MA, Novak L, et al. Risk model–guided clinical decision support for suicide screening: a randomized clinical trial. *JAMA Netw Open*. 2024;7(12):e2452371. doi:10.1001/jamanetworkopen.2024.52371

eFigure 1. Interruptive and NoniInterruptive CDS User Interfaces (Fake Patient Data Shown)

Figure 2. The CSSRS Custom Form (Excerpt) Developed for This RCT

eMethods. Seed Terms Derived From RCT Medical Record Review

eTable. Comments Input to CDS by Theme

This supplementary material has been provided by the authors to give readers additional information about their work.

© 2024 Walsh CG et al. *JAMA Network Open*.

eFigure 1. Interruptive and NoniInterruptive CDS User Interfaces (Fake Patient Data Shown)

A: Non-Interruptive CDS, “Elevated Suicide Risk” icon shown in patient panel

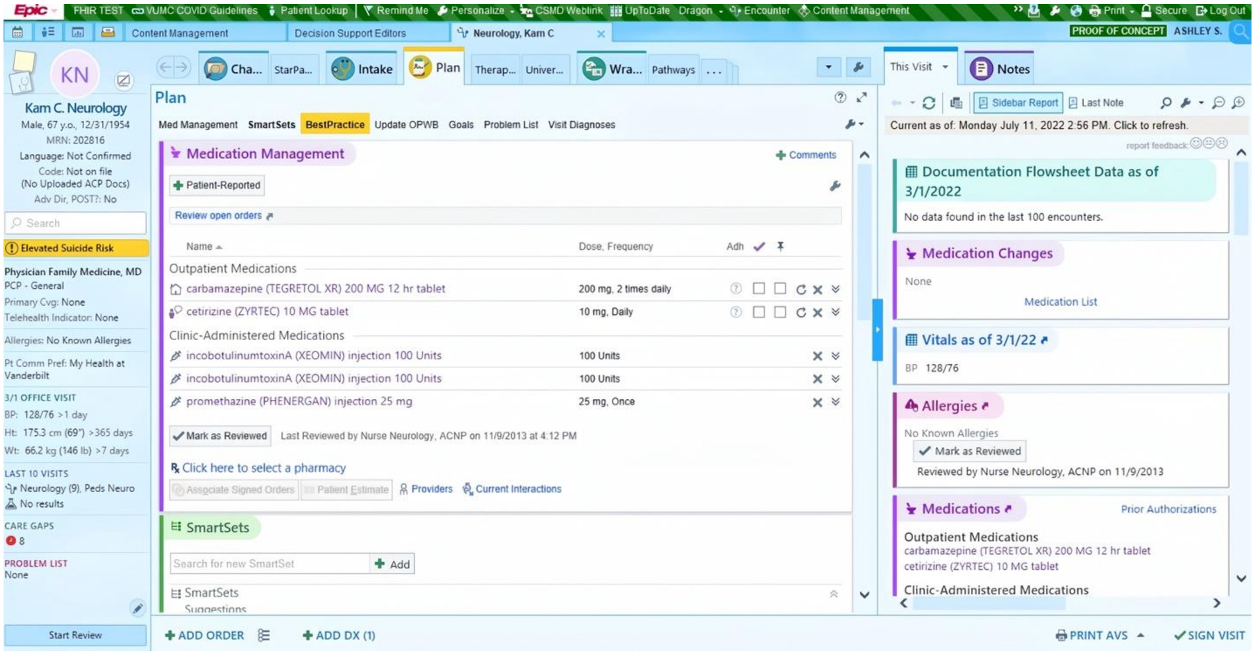

B. Noninterruptive CDS/Storyboard Icon Shown, Detail

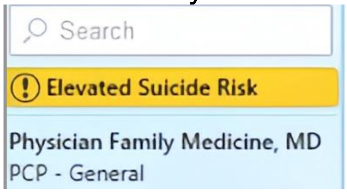

© 2024 Walsh CG et al. *JAMA Network Open*.

C: Interruptive CDS, BPA Highlighted (Orange) and Patient Panel Icon Highlighted (Purple)

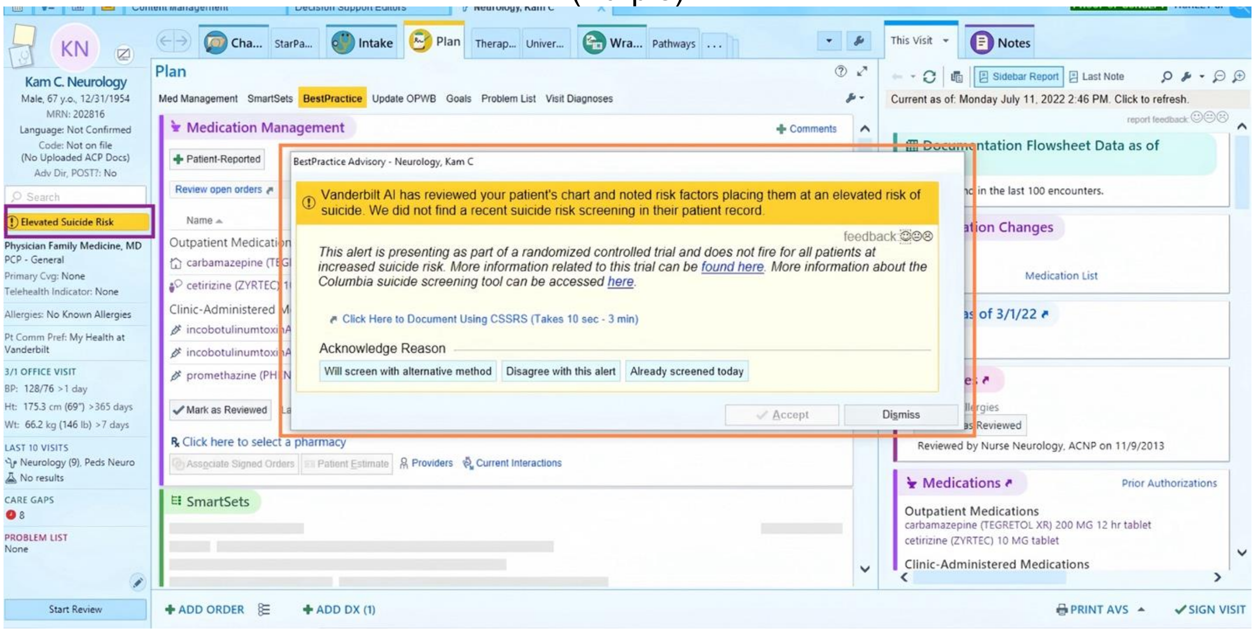

D: Interruptive CDS BPA shown, detail

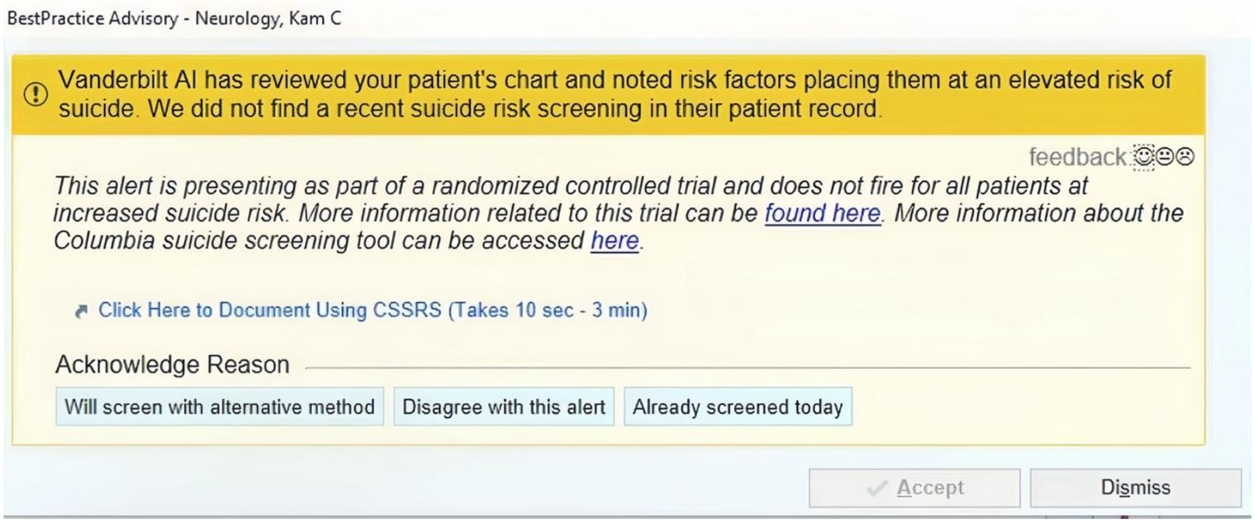

© 2024 Walsh CG et al. *JAMA Network Open*.

Figure 2. The CSSRS Custom Form (Excerpt) Developed for This RCT

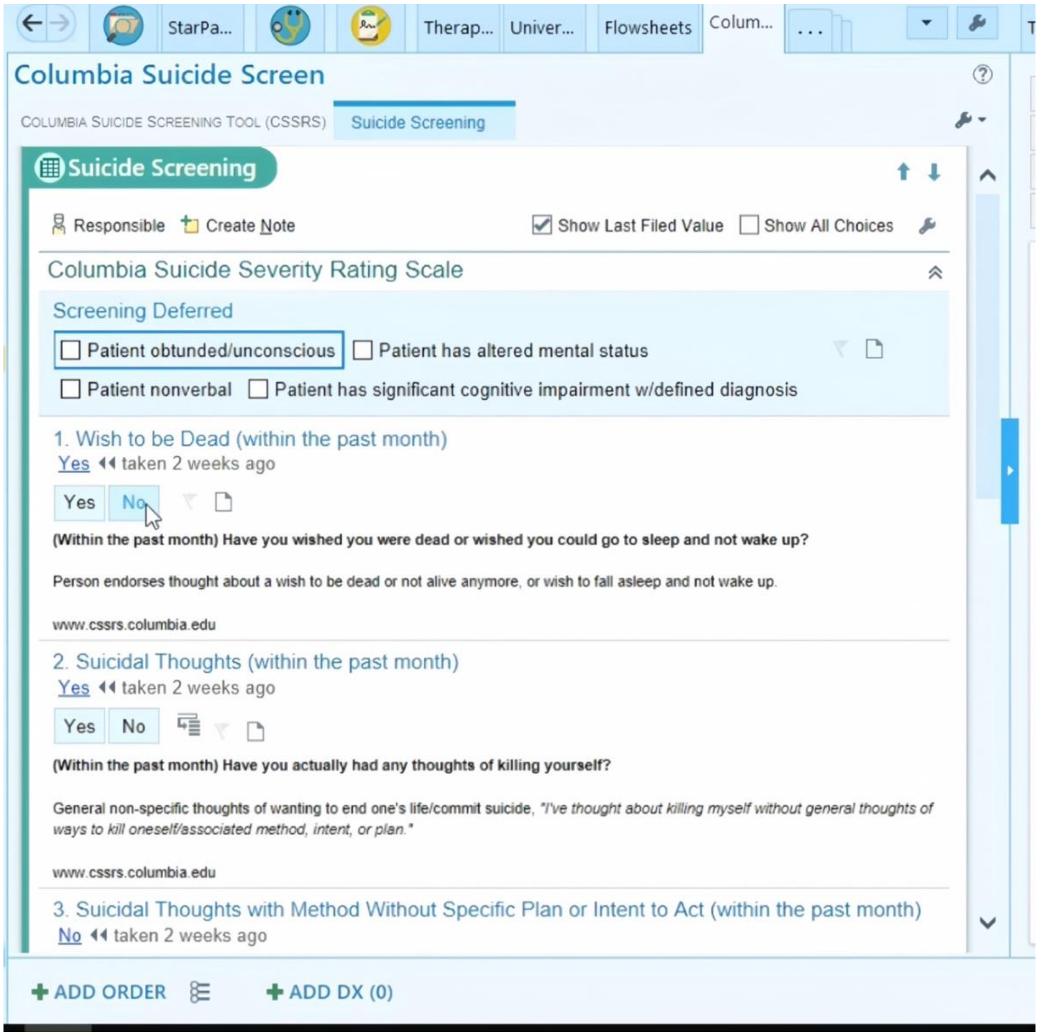

---

eMethods. Seed Terms Derived From RCT Medical Record Review
Seed terms extracted in RCT chart review with wildcards denoted as %: “suicid%”; “ SI.” “ SI“ “SI/HI” “phq” “cssrs”.

© 2024 Walsh CG et al. *JAMA Network Open*.

eTable. Comments Input to CDS by Theme

Theme	Comment
Screened (N=32)	administered PHQ-9, item #9 was 0; clinical interview prompted for SI/SA, denied by pt and informant
	BDI-II = 13 (minimal)
	C-SSRS administered - hard copy will be uploaded to EMR
	C-SSRS administered because of this alert
	C-SSRS performed because of this alert - form will be uploaded to EMR
	C-SSRS performed following alert trigger
	C-SSRS screen negative on day of encounter
	Denied current or past SI or suicidal behavior
	denied suicidal ideation on Beck Depression Inventory as well as on interview.
	Denies SI [two identical comments]
	gds15
	Interview
	Negative [two identical comments]
	No SI
	no SI at present
	no SI/HI
	Not suicidal
	phq9 [three identical comments]
	Reports no SI
	Screen negative
	Screened
	screened because of this alert
	screened day of visit
	Screened during appointment on <DATE>. Will be documented in neuropsych report
	Screened during clinical interview, No SI indiciated [sic].
	screened in part because of this alert
	screening initiated because of this alert
	will screen with C-SSRS, given this alert
Inappropriate for patient (N=4)	no risk factors [two identical comments]
	<Patient is non-verbal and "unable to express suicidality"... "I am uncertain of how to appropriately screen for suicidality">
	Patient with severe dementia
	inaccurate

© 2024 Walsh CG et al. *JAMA Network Open*.

Theme	Comment
Deferral (N=2)	I am opening the chart for the first time. I don't know anything about the pt
	patient seen with resident
Context (N=1)	did this the date of pt encounter; opening chart for addendum

Table: Physician comments to BPAs, [sic] noted and any study team redaction noted in <>

© 2024 Walsh CG et al. *JAMA Network Open*.
