# Supplementary material for: Risk Model–Guided Clinical Decision Support for Suicide Screening: A Randomized Clinical Trial
Source: JAMA Netw Open. 2025 Jan 3;8(1):e2452371. doi: 10.1001/jamanetworkopen.2024.52371 (PMC11699529; doi:10.1001/jamanetworkopen.2024.52371)
Supplement: Supplement 3. — Data Sharing Statement [file jamanetwopen-e2452371-s003.pdf]

Data Sharing Statement

Walsh. Risk Model–Guided Clinical Decision Support for Suicide Screening. *JAMA Netw Open*. Published January 03, 2025. doi:10.1001/jamanetworkopen.2024.52371

Data
Additional Information: Trial Registration ClinicalTrials.gov Identifier: NCT05312437
https://clinicaltrials.gov/study/NCT05312437
Data available: No

Additional Information
Explanation for why data not available: PHI/PII containing EHRs
